# Effects of Adaptive Non-linear Frequency Compression in Hearing Aids on Mandarin Speech and Sound-Quality Perception

**DOI:** 10.3389/fnins.2021.722970

**Published:** 2021-08-13

**Authors:** Shuang Qi, Xueqing Chen, Jing Yang, Xianhui Wang, Xin Tian, Hsuanyun Huang, Julia Rehmann, Volker Kuehnel, Jingjing Guan, Li Xu

**Affiliations:** ^1^Beijing Tongren Hospital, Capital Medical University, Beijing, China; ^2^Key Laboratory of Otolaryngology—Head and Neck Surgery, Beijing Institute of Otolaryngology, Capital Medical University, Ministry of Education, Beijing, China; ^3^Department of Communication Sciences and Disorders, University of Wisconsin—Milwaukee, Milwaukee, WI, United States; ^4^Division of Communication Sciences and Disorders, Ohio University, Athens, OH, United States; ^5^Sonova China, Shanghai, China; ^6^Sonova AG, Stäfa, Switzerland

**Keywords:** hearing aids, non-linear frequency compression, speech recognition, Mandarin Chinese, sound quality, acclimatization, adult

## Abstract

**Objective:**

This study was aimed at examining the effects of an adaptive non-linear frequency compression algorithm implemented in hearing aids (i.e., SoundRecover2, or SR2) at different parameter settings and auditory acclimatization on speech and sound-quality perception in native Mandarin-speaking adult listeners with sensorineural hearing loss.

**Design:**

Data consisted of participants’ unaided and aided hearing thresholds, Mandarin consonant and vowel recognition in quiet, and sentence recognition in noise, as well as sound-quality ratings through five sessions in a 12-week period with three SR2 settings (i.e., SR2 off, SR2 default, and SR2 strong).

**Study Sample:**

Twenty-nine native Mandarin-speaking adults aged 37–76 years old with symmetric sloping moderate-to-profound sensorineural hearing loss were recruited. They were all fitted bilaterally with Phonak Naida V90-SP BTE hearing aids with hard ear-molds.

**Results:**

The participants demonstrated a significant improvement of aided hearing in detecting high frequency sounds at 8 kHz. For consonant recognition and overall sound-quality rating, the participants performed significantly better with the SR2 default setting than the other two settings. No significant differences were found in vowel and sentence recognition among the three SR2 settings. Test session was a significant factor that contributed to the participants’ performance in all speech and sound-quality perception tests. Specifically, the participants benefited from a longer duration of hearing aid use.

**Conclusion:**

Findings from this study suggested possible perceptual benefit from the adaptive non-linear frequency compression algorithm for native Mandarin-speaking adults with moderate-to-profound hearing loss. Periods of acclimatization should be taken for better performance in novel technologies in hearing aids.

## Introduction

High-frequency components of acoustic signals convey useful information in speech and music. They play an important role in sound-quality perception, sound localization, speech perception in noise, and language development in children (Stelmachowicz et al., 2002, 2004; Monson et al., 2014; Moore, 2016). Many patients with sensorineural hearing loss have difficulty accessing high-frequency information. For this population, the most common intervention is to wear hearing aids. However, due to the limitation of audible bandwidth for speech information above 5 kHz in the conventional processing hearing aids (Boothroyd and Medwetsky, 1992; Moeller et al., 2007) and the presence of cochlear dead regions (Moore, 2001, 2004; Zhang et al., 2014), the aided performance in many hearing-aid users is not satisfactory. The frequency-lowering technique provides a practical solution because it shifts inaudible high frequencies to audible low-frequency regions (Simpson, 2009; Alexander, 2013; Mao et al., 2017). Among many different frequency lowering algorithms, non-linear frequency compression (NLFC) has been implemented in modern commercial hearing aids, such as Phonak Naida hearing aids. The key concept of NLFC is to disproportionally compress high frequencies into lower-frequency regions. In the first-generation of NLFC (known as SoundRecover or SR), two parameters, cut-off frequency (CT) and compression ratio (CR), determine the start point and strength of compression, respectively. Sound with frequencies below the CT remains unchanged but sound above the CT is compressed.

For people with severe-to-profound hearing loss, more aggressive settings with a lower CT and a higher CR are required because the patients have a narrower audible frequency bandwidth and the inaudible frequencies start at a lower frequency point in comparison to people with mild or moderate hearing loss. While the use of a lower CT ensures that a wider range of high frequencies can be shifted down so that they become audible to hearing aid users, it may also introduce unwanted detrimental effects to consonant and vowel perception (Alexander, 2016; Yang et al., 2018) and sound-quality perception (McDermott, 2011; Parsa et al., 2013; Souza et al., 2013). Therefore, to achieve a balance between audibility (lower CT) and fidelity (higher CT), Phonak introduced a new adaptive NLFC algorithm (known as SoundRecover 2 or SR2) in which the CT is switched between a low cut-off (CT1) and a high cut-off (CT2) based on the short-term energy distribution of the input signal (Rehmann et al., 2016). When the system detects sound energy at a relatively low-frequency region (e.g., vowels), CT2 is used so that the formants are not disturbed. When the incoming signal is a high-frequency sound (e.g., consonants), the system uses CT1. Technically, the adaptive NLFC preserves the spectral structure of vowel sounds and other low-frequency speech information and allows the accessibility of high-frequency information that is compressed and shifted to the lower-frequency region.

So far, there has been a number of studies examining the efficacy of NLFC on various aspects of speech perception including phoneme and word recognition, sentence perception, and sound-quality perception (Glista et al., 2009; Wolfe et al., 2010, 2011, 2017; Ching et al., 2013; Parsa et al., 2013; Brennan et al., 2014, 2017; Hopkins et al., 2014; McCreery et al., 2014; Picou et al., 2015; Alexander and Rallapalli, 2017; Chen et al., 2020; Xu et al., 2020). While many studies reported lower detection thresholds and improved perceptual accuracies with NLFC-fitted hearing aids in comparison to hearing devices fitted with conventional processing (CP) (Ching et al., 2013; Alexander et al., 2014; Zhang et al., 2014; Ching and Rattanasone, 2015), some studies reported no additional benefit in phoneme audibility, or sentence recognition with the NLFC algorithm (Perreau et al., 2013; Bentler et al., 2014; Picou et al., 2015). In addition, within those studies that found improved perceptual performance with NLFC, some reported that the benefit of NLFC was not ubiquitously shown in all tested subjects (Simpson et al., 2005; Glista et al., 2009; McCreery et al., 2014). For example, Simpson et al. (2005) tested the recognition of monosyllabic words with NLFC vs. CP in 17 participants with sloping moderate-to-severe sensorineural hearing loss. Only eight of them showed improved recognition accuracy and one participant demonstrated decreased accuracy with NLFC compared to CP. As summarized in Akinseye et al. (2018), there still lacks convincing evidence supporting the superiority of NLFC over CP in all hearing-related tasks.

As adaptive NLFC is a newly developed algorithm, only a few studies tested the use of this algorithm in hearing-impaired listeners (e.g., Glista et al., 2017, 2019; Xu et al., 2020). In these studies, the researchers compared the perceptual performance with CP, NLFC, and adaptive NLFC of the tasks including phoneme perception, word recognition, and sound-quality ratings in hearing-impaired children and/or adults. Wolfe et al. (2017) reported a lower threshold for phoneme detection and higher accuracy for phoneme and word recognition in the tested children. Glista et al. (2017) found that both NLFC and adaptive NLFC provided greater benefits on phonetic recognition than CP. However, there was no significant difference between static NLFC and adaptive NLFC on phoneme perception. In a recent study, Xu et al. (2020) evaluated the efficacy of the adaptive NLFC (i.e., SR2) on phoneme detection, speech detection threshold, and sound-quality ratings in Mandarin-speaking hearing-impaired adults. In that study, five SR2 settings (SR2-off, SR2-default, SR2-weak, SR2-strong 1, and SR2-strong 2) with various fitting parameters for CT1, CT2, and CR were compared. The results revealed that the hearing-impaired listeners showed improved (lowered) phoneme detection and speech detection thresholds with the two strong settings than weak setting or off condition. However, different settings did not exert a significant influence on sound-quality ratings. While Xu et al. (2020) study focused on detection ability through one-time tests, the current study aimed to provide a more comprehensive evaluation to test the impact of adaptive NLFC on different aspects of perception ability including Mandarin consonant, vowel, and sentence recognition. Additionally, because each participant was tested multiple times spanning a 3-month period, the design of this study enabled us to examine how the perceptual performance would change as a function of increased experience with adaptive NLFC-fitted hearing aids.

## Materials and Methods

### Participants

**FIGURE 1:**
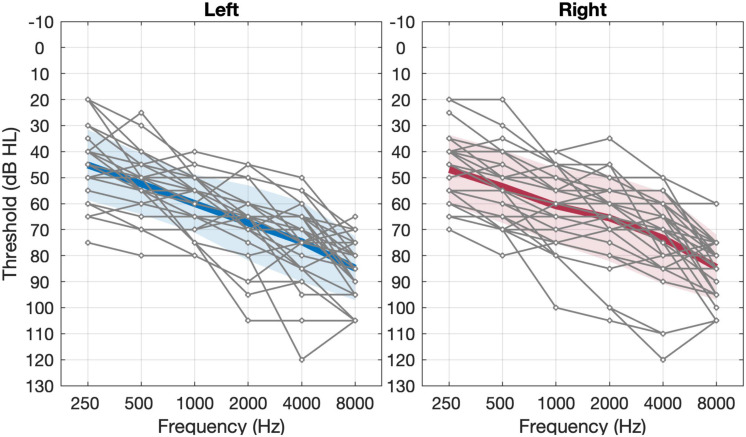
Unaided pure-tone thresholds of **left** and **right** ears in the 29 subjects. The thin gray lines represent individual thresholds and the thick black line represents the group mean threshold (*n* = 29). The shaded area represents ±1 S.D. of the mean.

The participants included 29 Mandarin-speaking adults (13 females and 16 males) aged between 37 and 76 years old (*M* = 66.7, *SD* = 8.8). All participants were diagnosed with sloping moderate-to-profound sensorineural hearing loss. The average pure-tone audiometric thresholds between 500 and 4,000 Hz for both ears were between 40 and 90 dB HL. The individual and group average pure-tone thresholds are shown in Figure 1. The duration of hearing loss ranged from 2 to 40 years, with a mean of 13.1 years. All participants met the following recruitment criteria: (1) symmetric sloping sensorineural hearing loss (i.e., interaural difference ≤ 15 dB at all octave frequencies from 250 to 8,000 Hz) with air-bone gaps at any frequency ≤ 15 dB; (2) normal middle ear function as indicated by tympanometry and otoscopy examinations; (3) no diagnosed cognitive or mental impairments (able to communicate effectively with their families and the investigators); (4) no experience of hearing aids with frequency lowering schemes prior to participating in the present study; and (5) native Mandarin speakers in daily life. Twenty-two of the participants had used hearing aids before participating in the study whereas seven had no hearing aid experience. All 29 participants completed all five test sessions. This study protocol was reviewed and approved by the Institutional Review Boards of Ohio University and Beijing Tongren Hospital.

### Hearing Aid Fitting

All participants were bilaterally fitted with experimental hearing aids (Phonak Nadia V90-SP) programmed in Phonak’s Target fitting software (v. 5.1). To ensure proper amplification of sound across the entire speech spectrum and to limit acoustic feedback, occluding hard ear-molds made of acrylic materials were used with different vent sizes based on the recommendation of the fitting software. After the feedback and real-ear test using the estimated RECD (real-ear-to-coupler difference) and recommended vents, the APDT (Adaptive Phonak Digital Tonal) gain algorithm was chosen as the prescriptive target. The output gain level was initially set to 100%, decreasing in a 10% step size in case the participant reported that the hearing aids were too loud. Three SR2 settings were tested in the study: SR2 off, SR2 default, and SR2 strong (i.e., moving three steps toward Audibility relative to default on the upper slider). As a group, the parameters CT1 and CT2 changed from 3.77 ± 1.35 (mean ± SD) and 5.12 ± 1.21 kHz in SR2 default to 2.17 ± 0.55 and 3.78 ± 0.67 kHz in SR2 strong. The group average of the parameter CR remained unchanged in SR2 default and SR2 strong (1.22 ± 0.10 vs. 1.22 ± 0.06). Note that the use of lower CT1 and CT2 in SR2 strong could potentially move more high-frequency energy to the audible range but create greater disruption of low-frequency information. A schematic diagram of the signal processing for SR2 is available in our previous acoustic study of non-linear frequency compression (See Figure 1 of Yang et al., 2018). The other advanced functions (such as noise reduction, directionality, etc.) were all set as default. All the adjustments were performed by experienced audiologists. Moreover, all the settings embedded in the hearing aids remained the same throughout the process of the entire study. All participants wore the same experimental hearing aids with only setting varied (see Procedures below) throughout the study period.

### Perceptual Tasks and Outcome Measures

The perceptual performance of each participant was evaluated through speech perception tests and sound-quality rating tasks. The speech perception tests included Mandarin-Chinese consonant, vowel, and sentence recognition tests.

#### Consonant Recognition

The consonant recognition test included five Mandarin fricatives (i.e., f /f/, s /s/, x /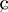/, sh /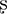/, and h /x/) and six Mandarin affricates (i.e., z /ts/, c /ts^h^/, j /t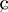/, q /t
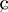
^h^/, zh /t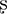/, ch /t
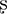
^h^/) embedded in a /Ca/ syllable in tone 1. The 11 words containing the target consonants are 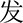 (fā), 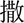 (sā), 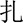 (zā), 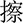 (cā), 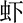 (xiā), 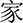 (jiā), 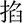 (qiā), 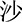 (shā), 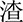 (zhā), 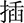 (chā), and 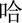 (hā). The tokens were recorded from 6 adult Mandarin speakers (3 males and 3 females). Thus, the consonant recognition test comprised 66 tokens (11 words × 6 speakers) that were randomly presented to the participants. The intensity of the stimuli was set at 65 dB SPL.

#### Vowel Recognition

The Mandarin vowel list included 12 Mandarin vowels (i.e., /a/, /ai/, /ao/, /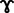/, /i/, /iao/, /ie/, /iou/, /ou/, /u/, /uei/, /uo/) embedded in a /dV/ syllable structure in tone 1. The 12 monosyllabic words are 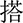 (dā), 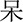 (dāi), 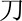 (dāo), 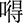 (dē), 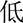 (dī), 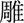 (diāo), 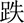 (diē), 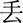 (diū), 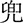 (dōu), 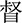 (dū), 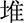 (duī), and 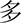 (duô). The tokens were recorded from the same 6 adult Mandarin speakers. In total, there were 72 tokens for the vowel recognition test (12 words × 6 speakers). The intensity of the stimuli was also set at 65 dB SPL.

#### Sentence Recognition

The material used for sentence recognition was Mandarin Hearing in Noise Test (MHINT) (Wong et al., 2007). MHINT contains 12 sentence lists. Each list is composed of 20 sentences each of which is 10 Chinese characters long. The intensity of the sentence stimuli was fixed at 65 dB SPL. In order to reduce the ceiling effect for sentence recognition, a speech-spectrum-shaped noise (Xu et al., 2021) was mixed with the sentences at a signal-to-noise ratio (SNR) of +5 dB. For each SR2 setting, participants were tested with one different list randomly chosen from the 12 sentence lists. A total of 11 lists were used throughout the five sessions in which two lists were used for sessions 1, 2, 4, and 5 and three lists were used for session 3 (see below in Procedures section for details on test sessions and SR2 conditions). The final score was calculated based on the percent of correct characters in each test list.

#### Sound-Quality Rating

**FIGURE 2:**
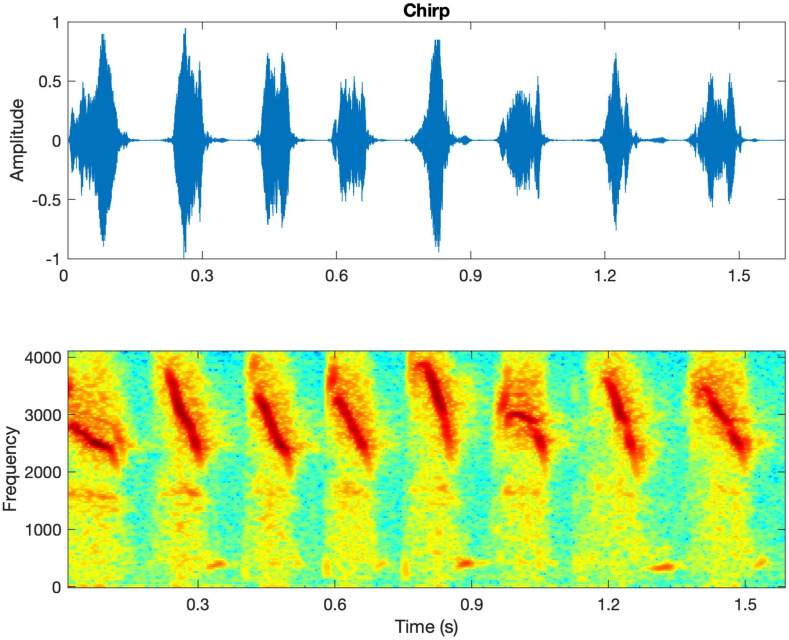
The waveform and spectrogram of the bird chirps. The spectrogram shows that the chirps are rapid downward frequency sweeps from 4000 to 2,000 Hz approximately.

All participants were asked to rate the loudness, clarity, naturalness, and overall sound quality of different types of sounds including own voice, male voice, female voice, bird chirp, and music. Own voice was referred to as the participant’s natural and spontaneous vocal production in daily life after wearing the experimental hearing aids. The male voice and female voice were reading text composed of 127 Chinese characters by one male and one female talker. The lengths of the recordings were 34 and 36 s for the male and female voices, respectively. They were presented at 65 dB(A) in quiet. The bird chirps were provided by MATLAB software (MathWorks, Natick, MA). The original 8 chirps (Figure 2) were repeated twice to form 24 chirps with a total duration of 5.5 s. The music was a piece of recorded piano music excerpted from a classic and well-known Chinese folk music entitled “Liang Zhu (The Butterfly Lovers).” The bird chirps and music were presented at 65 and 70 dB(A), respectively.

The five types of stimuli were presented in a fixed order as above with no repetition being allowed. After listening to each stimulus, participants were asked to rate four aspects of sound quality including loudness, clarity, naturalness, and overall quality using a continuous bar with two ends being 0 (extremely poor) and 10 (perfect). No practice was provided for this task.

### Procedures

**FIGURE 3:**
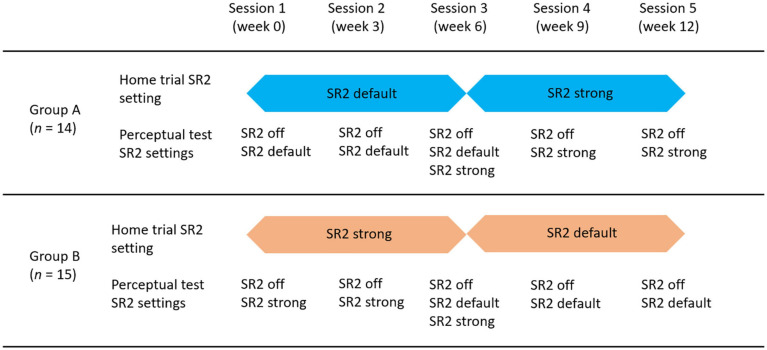
Diagram of test procedures and group assignments. There were 5 sessions equally distributed in the 12-week period. Participants were randomly assigned into two groups. Group A (*n* = 14) used SR2 default as the home trial setting for the first 6 weeks and switched to SR2 strong for the home trial in the second 6 weeks. Group B (*n* = 15) used SR2 strong for the first 6 weeks and SR2 default for the second 6 weeks in their home trial.

All participants were tested at five different sessions separated by 3 weeks between each two consecutive sessions. In the first session, the participants were fitted with bilateral hearing aids (Phonak Nadia V90-SP BTE) with individualized hard ear-molds. The sound-field aided thresholds with SR2 off, SR2 default, and SR2 strong settings were measured using warble tones at 250, 500, 1,000, 2,000, 3,000, 4,000, 6,000, and 8,000 Hz. Perceptual tests including speech perception (i.e., consonant, vowel, and sentence recognition) and sound-quality rating with SR2 off and SR2 default or SR2 strong settings were then conducted. After the first session, the participants were sent home with the hearing aids on either SR2 default or SR2 strong. To count-balance the order of SR2 settings, 14 of the 29 participants wore SR2 default for the first 6 weeks (Group A) and the remaining 15 of the 29 participants wore SR2 strong for the first 6 weeks (Group B). After 6 weeks, the SR2 settings for both groups were switched. Figure 3 illustrates the SR2 settings used in the home trial and those used in the perceptual tests in the lab. The order of SR2 setting used in the perceptual tests was randomized across participants and test sessions. All tests were conducted in a sound booth, with the background noise below 30 dB A. The stimuli were presented through a loudspeaker located at 1.45 m in front of the participants at 0° azimuth.

### Statistical Analyses

Statistical analyses were conducted using R software (version 3.63). The percent-correct data of the recognition tests were treated as binomial data (Thornton and Raffin, 1978). A generalized linear mixed-effect model (GLMM) (Warton and Hui, 2011) was used to investigate the impacts of (1) SR2 settings (off, default, and strong) and (2) test sessions on the percent-correct scores. Furthermore, we analyzed the potential interactions between SR2 setting and test session. For the sound-quality rating data, linear mixed models (LMM) were performed separately for each category of sound-quality percept (i.e., loudness, clarity, naturalness, and overall preference). The three main factors were (1) SR2 settings, (2) test sessions, and (3) sound types (i.e., own voice, male voice, female voice, bird chirp, and music).

## Results

### Group Aided Thresholds

**FIGURE 4:**
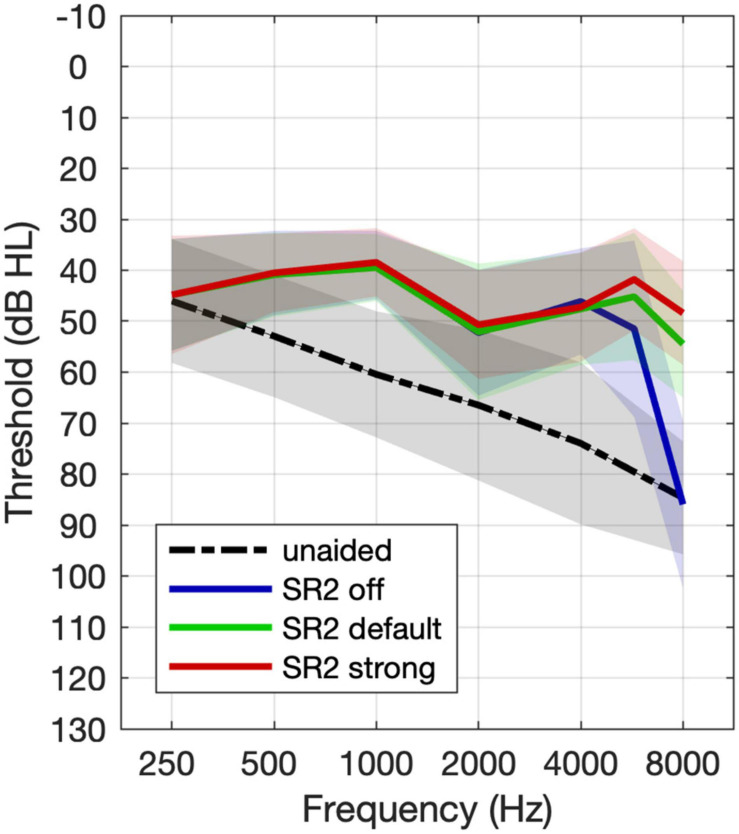
Mean group unaided and aided hearing thresholds. The black line represents the unaided thresholds. The dashed line, the solid line, and the dotted lines represent free-field thresholds for the aided thresholds with SR2 off, SR2 default, and SR2 strong settings, respectively. The shaded area represents ±1 S.D. of the mean.

The group mean unaided and aided hearing thresholds under the three SR2 settings (Figure 4) were analyzed using repeated-measures analysis of variance (ANOVA) by frequency separately. Compared with unaided thresholds, aided hearing thresholds were better at all frequencies (with *F*-values ranging from 8.87 to 77.92, all *p* < 0.001) except 250 Hz [*F*_(3,__118)_ = 0.07, *p* = 0.98]. Tukey-adjusted pairwise comparisons revealed no significant differences in aided hearing thresholds among SR2 off, SR2 default, and SR2 strong settings from 250 Hz to 6 kHz (all adjusted *p* > 0.05). At 8 kHz, the hearing thresholds for SR2 off setting and unaided condition were comparable (adjusted *p* = 0.34), and those for SR2 default and SR2 strong settings were not significantly different (adjusted *p* = 0.29). However, the hearing threshold was significantly higher at for unaided or SR2 off setting than for SR2 default setting (unaided vs. SR2 default: adjusted *p* < 0.0001; SR2 off vs. SR2 default: adjusted *p* < 0.0001) and SR2 strong setting (unaided vs. SR2 strong: adjusted *p* < 0.0001; SR2 off vs. SR2 strong: adjusted *p* < 0.0001).

### Speech Perception Tests

**FIGURE 5:**
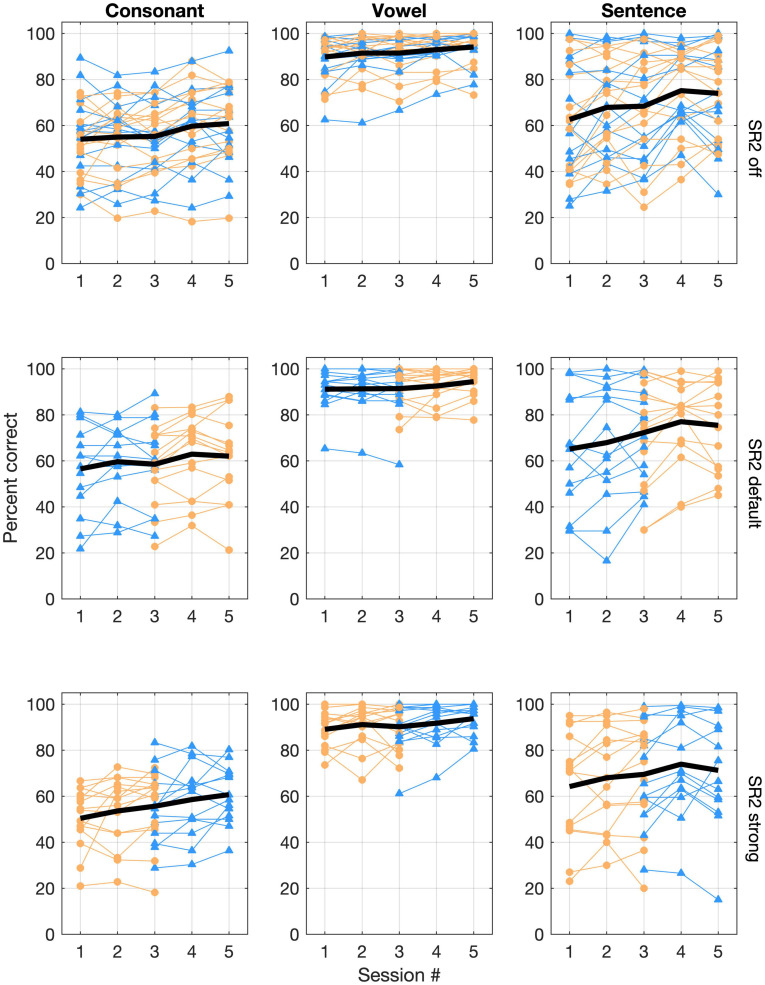
Individual and group mean speech-recognition performance. Consonant and vowel recognition in quiet, and sentence recognition in noise (+5 dB SNR) are represented in the three columns whereas performance for the three SR2 settings is represented in the three rows of panels. In each panel, performance scores (% correct) are plotted as a function of test sessions. Each thin line represents one participant. Those in Group A are plotted with triangles in blue whereas those in Group B are plotted with circles in orange. The thick black line represents the overall group mean performance.

Individual and group average performance of consonant, vowel, and sentence recognition with the three SR2 settings in five sessions is shown in Figure 5. All participants underwent a 12-week trial of the two SR2-enabled settings (i.e., SR2 default and SR2 strong), each of which for 6 weeks, respectively. As explained in Figure 3 and associated text, 14 of the 29 participants (Group A) used SR2 default setting in the first 6 weeks, while the other 15 participants (Group B) used SR2 strong setting first. In the sixth week, the two SR2 settings were switched. This counterbalance design minimized potential order effects. As shown in Figure 5, no apparent order effects were observed in the speech recognition data. An independent *t*-test comparing the recognition performance between Groups A and B revealed no order effect [consonant recognition: *t*(27) = −0.57, *p* = 0.57; vowel recognition: *t*(27) = −1.17, *p* = 0.24; sentence recognition: *t*(27) = −0.95, *p* = 0.35]. Thus, in the following presentations of results, data from Groups A and B were pooled together and were not treated separately.

Large individual variability in speech recognition, especially in consonant recognition in quiet and sentence recognition in noise (+5 dB SNR), was evident (Figure 5). Pearson correlation analyses showed that consonant recognition scores in the three SR2 settings were correlated with the participants’ aided thresholds (with corresponding SR2 settings) at high frequencies (i.e., 4,000, 6,000, or 8,000 Hz) as well as averaged thresholds across 500, 1,000, 2,000, and 4,000 Hz (PTA_250–4,000 *Hz*_) (correlation coefficients ranging from −0.411 to −0.741, *z*-test, all *p* < 0.05) with exception of one condition [i.e., 8,000 Hz with the SR2 strong setting (*r* = −0.311, *p* = 0.1)]. In addition, we used the difference scores in speech recognition between SR2 default and SR2 off settings or those between SR2 strong and SR2 off settings as potential NLFC benefit scores. However, Pearson correlation analyses revealed no significant correlation of the latter and the participants’ aided hearing thresholds at high frequencies or PTA_250–4,000 *Hz*_. We also found that the participants’ age was not correlated with any of the speech recognition performance nor the potential NLFC benefit scores.

**FIGURE 6:**
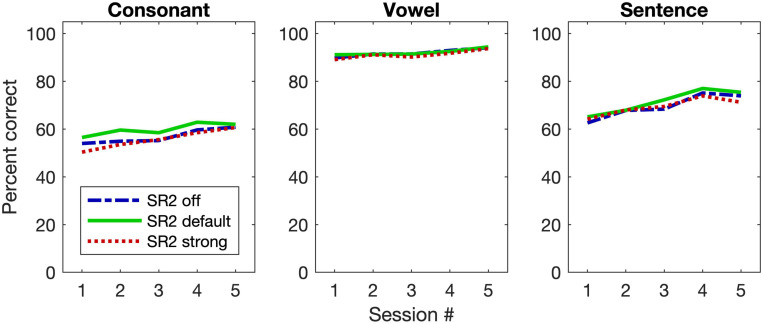
Group mean performance of speech-recognition performance. The three panels are for consonant and vowel recognition in quiet, and sentence recognition in noise (+5 dB SNR), respectively. The three different lines represent the group mean results for the three SR2 settings.

Figure 6 plots the group mean speech-recognition results of the three SR2 settings as a function of the test session. For consonant recognition, the GLMM analysis revealed that both SR2 settings (i.e., SR2 off, SR2 default, and SR2 strong) and test sessions (i.e., 1, 2, 3, 4, and 5) were significant factors for the recognition performance [SR2 settings: χ^2^(2, *N* = 29) = 21.90, *p* < 0. 0001; sessions: χ^2^(4, *N* = 29) = 68.95, *p* < 0.0001]. *Post-hoc* multiple comparisons revealed a modestly better performance for SR2 default setting than for SR2 off setting (adjusted *p* = 0.001) or SR2 strong setting (adjusted *p* < 0.001). No significant differences between SR2 off and SR2 strong settings were observed (adjusted *p* = 0.41). The participants also showed improved performance from Session 1 to Session 5. Multiple comparisons revealed that the participants performed similarly at Sessions 4 and 5 (adjusted *p* = 0.98) which were both significantly better than at Sessions 1, 2, and 3 (all adjusted *p* < 0.001). The interaction between test session and SR2 setting was not significant [χ^2^(8, *N* = 29) = 4.31, *p* = 0.83].

For vowel recognition, unlike consonant recognition, only test session contributed to the improved performance [χ^2^(4, *N* = 29) = 61.73, *p* < 0.00]. Specifically, the vowel recognition at Session 5 was significantly better than that at Sessions 1, 2, and 3 (all adjusted *p* < 0.001). Between Sessions 4 and 5, however, no significant differences were observed (adjusted *p* = 0.006). This was also true for the pairwise comparisons among Sessions 1, 2, and 3 (all adjusted *p* > 0.1).

For sentence recognition in noise, similar to consonant perception, both test sessions and SR2 settings contributed to the improved performance [session: χ^2^(2, *N* = 29) = 19.96, *p* < 0.0001; SR2 setting: χ^2^(4, *N* = 29) = 302.22, *p* < 0.0001] and no interaction was found between the two factors [χ^2^(8, *N* = 29) = 8.27, *p* = 0.41]. The sentence-recognition performance for SR2 default setting was the highest as compared to SR2 off setting (adjusted *p* = 0.001) or SR2 default setting (adjusted *p* = 0.003). No difference between SR2 off and strong was found (adjusted *p* = 0.99).

**FIGURE 7:**
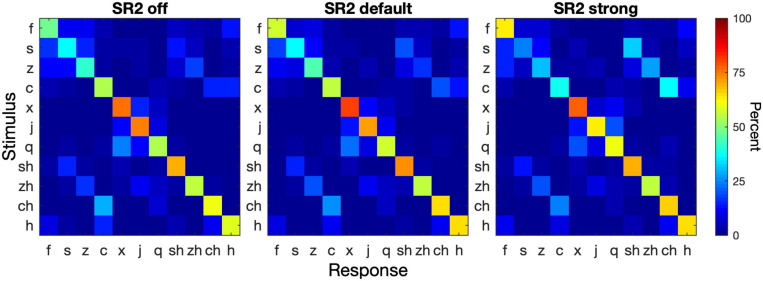
Consonant confusion matrices. Consonant recognition data were collapsed from all 5 sessions. The three panels show the confusion matrices for SR2 off, SR2 default, and SR2 strong settings. In each panel, the stimulus is represented by the ordinate and the response by the abscissa. The color of each cell in a matrix represents the percent of a stimulus being identified as a particular consonant (see color bar on the right).

Consonant confusion analyses were then conducted to illustrate potential confusion patterns that might help to explain the modest improvement with the frequency-lowering technique. Figure 7 shows the consonant confusion matrices averaged across all 5 sessions. Of all tested consonants, the alveolar sounds /s, ts, ts^h^/ (s, z, c) showed the lowest recognition accuracy and alveolopalatal sounds /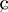, t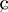, t
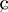
^h^/ (x, j, q) showed the highest accuracy. Among the five places of articulation, confusions mainly occurred between the alveolar sounds /s, ts, ts^h^/ (s, z, c) and the retroflex postalveolar /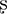, t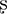, t
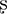
^h^/ (sh, zh, ch), which showed an asymmetrical pattern. That is, the alveolar sounds were more likely to be recognized as the retroflex sounds, rather than the other way around. Additionally, as the SR setting became stronger, the degree of confusion of alveolar sounds as retroflex sounds increased.

### Sound-Quality Rating

**FIGURE 8:**
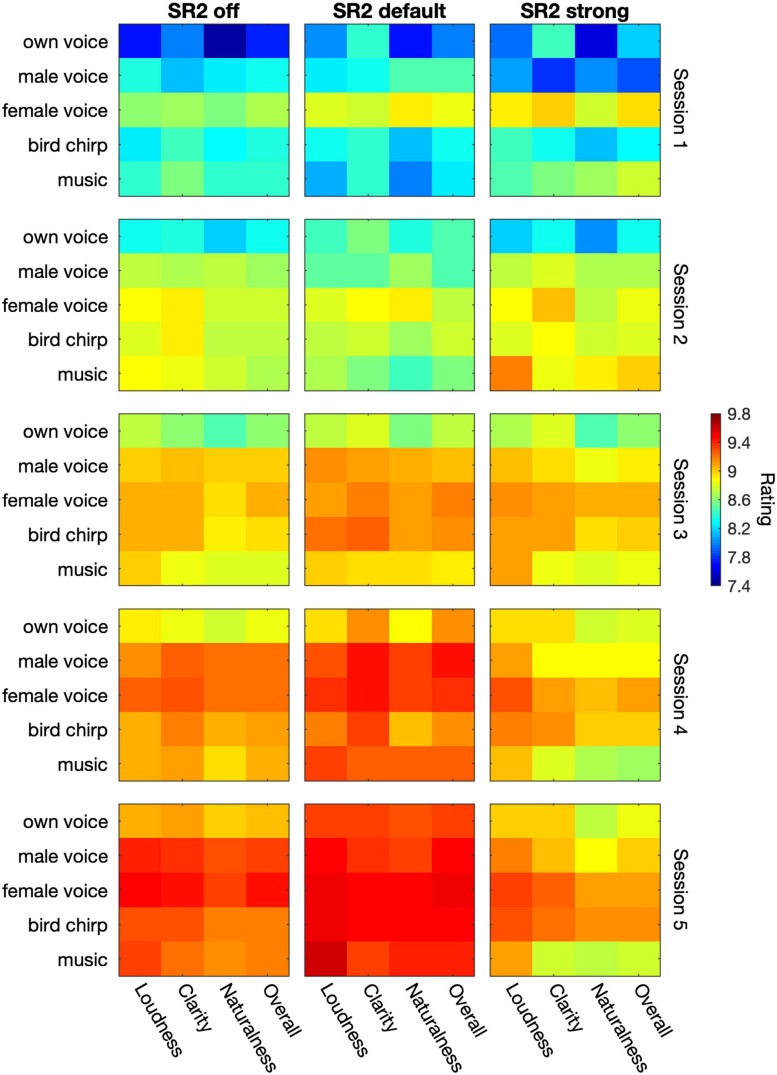
The average sound-quality ratings from the 29 participants. The three columns represent SR2 off, SR2 default, and SR2 strong setting, respectively. From top to down, the 5 rows indicate the 5 sessions. In each panel, the five types of stimuli (i.e., own voice, male voice, female voice, bird chirps, and music) are represented in rows whereas the category of percepts (i.e., loudness, clearness, naturalness, and overall quality) are represented in columns. The color in each cell represents the rating score as indicated by the color bar on the right.

Figure 8 plots the average sound-quality ratings from the 29 participants in the three SR2 settings and five test sessions. The LMM analyses revealed different patterns in each category of sound-quality percept (loudness, clarity, naturalness, and overall preference). For the rating of loudness, the analysis yielded significant main effects of test session [*F*_(__4__, 1555__.6)_ = 106.44, *p* < 0.0001] and type of stimulus [e.g., own voice, male, female, bird chirps, and music, *F*_(__4__, 1553__.8)_ = 25.7, *p* < 0.0001]. SR2 settings [*F*_(__2__, 1554__.4)_ = 1.01, *p* = 0.36] as well as the interaction between test session and SR2 setting [*F*_(__8__, 1558__.4)_ = 0.38, *p* = 0.93] were not significant. The participants’ satisfaction with loudness improved progressively from Session 1 to Session 5 (all adjusted *p* < 0.001). Among the five different types of stimuli, the rating score for own voice was significantly lower than all the other four types of stimuli (all adjusted *p* < 0.001). In addition, the overall quality rating for the female voice was found to be higher than for the male voice (adjusted *p* = 0.028). No other significant differences were observed (all adjusted *p* > 0.05).

In terms of clarity rating, all the three main factors were significant [test session: *F*_(__4__, 1558__.9)_ = 4.10, *p* = 0.016; SR2 setting: *F*_(__2__, 1557__.6)_ = 71.16, *p* < 0.0001; type of stimulus: *F*_(__4__, 1557__)_ = 18.59, *p* < 0.0001] but there was no interaction between test session and SR2 setting [*F*_(__8__, 1561__.7)_ = 0.72, *p* = 0.68]. The participants rated clarity significantly higher with SR2 default setting than with SR2 strong setting (adjusted *p* = 0.018), while no difference was detected between SR2 off and SR2 default settings (adjusted *p* = 0.17) or between SR2 off and SR2 strong settings (adjusted *p* = 0.041). The clarity rating at Sessions 4 and 5 was the highest compared to that at Sessions 1, 2, and 3 (all adjusted *p* < 0.001), yet no significant difference was observed at Session 5 compared with Session 4 (adjusted *p* = 0.59). Finally, own voice clarity scores were found to be the lowest among all types of stimuli (all adjusted *p* < 0.001). The female voice, on the other hand, received rating scores significantly higher than the male voice (adjusted *p* < 0.001) or music (adjusted *p* = 0.001) but similar scores to the chirp (adjusted *p* = 0.39). All other pairwise comparisons were not significant otherwise (all adjusted *p* > 0.1).

For the naturalness and overall rating, all the three main factors (i.e., SR2 setting, test session, and type of stimulus) played significant roles in the outcome measures [Naturalness: *F*_(__2__, 1557__.7)_ = 6.14, *p* = 0.002; *F*_(__4__, 1558__.9)_ = 78.00, *p* < 0.0001; *F*_(__4__, 1557__.1)_ = 34.53, *p* < 0.0001; Overall: *F*_(__2__, 1557__.7)_ = 6.39, *p* = 0.0017; *F*_(__4__, 1558__.9)_ = 78.15, *p* < 0.0001; *F*_(__4__, 1557__.1)_ = 24.80, *p* < 0.0001] but not the interaction terms [*F*_(__8__, 1561__.6)_ = 0.86, *p* = 0.55; *F*_(__8__, 1561__.7)_ = 0.60, *p* = 0.78]. Both naturalness and overall ratings improved from Session 1 to 4 (naturalness: all adjusted *p* < 0.0001; overall quality: all adjusted *p* < 0.0001) but not from Session 4 to 5 (naturalness: adjusted *p* = 0.27; overall quality: adjusted *p* = 0.11). Both ratings were found to be better for SR2 default setting than for SR2 strong setting (both adjusted *p* = 0.002), but no difference was found for the other comparisons among SR2 settings. Among different types of stimuli, own voice was rated with the lowest scores for both naturalness (all adjusted *p* < 0.0001) and overall quality scores (all adjusted *p* < 0.0001). In addition, the female voice was rated with significantly higher naturalness than music (adjusted *p* = 0.009) and with higher overall quality scores than the other types of stimuli (all adjusted *p* < 0.05).

## Discussion

In the present study, we evaluated the efficacy of the new adaptive NLFC scheme on Mandarin speech perception and sound-quality ratings in adult hearing aid users. A series of tests were conducted over a 3 month period to evaluate aided hearing thresholds, consonant and vowel recognition in quiet, sentence recognition in noise, and subjective sound-quality ratings in 29 participants with severe-to-profound hearing impairment.

For sound detection, our results demonstrated an improvement of more than 30 dB in detecting high-frequency sounds at 8 kHz with the application of adaptive NLFC scheme (Figure 4). A similar tendency was found for 6 kHz but the change of the detection threshold with the adaptive NLFC on vs. off was only 5 dB. This change was not statistically nor clinically significant. The reduced effect of SR2 on the detection of the 6-kHz tone in the present study may be due to the fact that the hearing loss at 6 kHz for most of our participants was not too severe (Figure 1) and that the hearing aids provided adequate amplification at that frequency even with the conventional processing scheme (Figure 4). Nonetheless, the detection results were consistent with findings by Xu et al. (2020) in which a group of 15 adult hearing-aid users with sloping severe-to-profound sensorineural hearing loss showed substantial improvement in detecting high-frequency Mandarin phonemes such as /s/ (centered at both 6 and 9 kHz) and /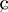/ (“x”), especially with the stronger SR2 settings.

For vowel recognition, most of the participants had recognition scores above 90% correct (Figure 5). The adaptive NLFC scheme did not show a significant impact on the recognition performance in this experiment. This result was similar to previous findings in vowel recognition using the static NLFC scheme (i.e., SR) (Yang et al., 2018; Chen et al., 2020). The first three formants that characterize vowel identity reside in relatively low frequency regions, usually lower than 4 kHz. In contrast to high-frequency sounds, vowel sounds are typically accessible in patients with sensorineural hearing impairments. SR2, even with a strong setting, enables well-preserved formant patterns due to the application of two cutoffs. In the current study, even under the SR2 strong setting, the average CT2 (3.78 kHz) is out of the range of the second and third formants for most vowels (Alexander, 2016). With the application of adaptive cutoffs, the formant structure of Mandarin vowel sounds was preserved well and vowel recognition accuracy remained very high. Another possible reason could be that Mandarin has a small inventory of monophthongal vowel phonemes and less crowded vowel space in comparison to many languages such as English. Even though the compression process might modify the spectral features of certain vowels [e.g., F2 of Mandarin high front vowel /i/ as shown in Yang et al. (2018)], the distorted spectral structure introduced a limited detrimental effect on recognition. Therefore, Mandarin vowel recognition was not negatively affected by the adaptive NLFC scheme, even in the strong setting.

Unlike vowel recognition, the use of adaptive NLFC significantly improved consonant recognition performance. Chen et al. (2020) reported improved Mandarin consonant recognition with SR default setting. In the present study, we observed improved accuracy for consonant recognition with SR2 default in comparison to SR2 off. It is noteworthy that the participants’ average hearing loss in the present study was more severe at 4 and 8 kHz than that in the previous SR study by Chen et al. (2020). The unaided thresholds at 4 and 8 kHz were approximately 10 dB higher in the present study than those in Chen et al. (2020). The significant improvement of consonant recognition with SR2 default setting in patients with more severe hearing loss suggests a possible application of this setting in patients with a wider range of hearing loss. While the SR2 default setting significantly improved the recognition accuracy, a stronger setting did not improve the consonant recognition performance further. This finding is similar to the outcome reported in Xu et al. (2020) that a stronger compression does not ensure better recognition performance. A possible explanation is that strong compressions might cause more confusion for high-frequency consonants and thus offset the benefit of better detection. When the incoming signals contain predominantly high-frequency energy, as in the consonants, CT1 (with higher compression) will be applied. In this study, the average CT1s were 3.77 kHz (SR2 default) and 2.17 kHz (SR2 strong), suggesting that a lower cut-off frequency for SR2 could limit recognition performance. The better performance with SR2 default than SR2 strong suggested that there might be an optimal range of frequency compression for individuals with high-frequency hearing loss, as suggested by several previous studies (Johnson and Light, 2015; Scollie et al., 2016; Glista and Scollie, 2018).

Our consonant confusion analyses (Figure 7) showed that not all consonants were equally affected by NLFC. The positive effects of NLFC on recognition of certain Mandarin consonants was negated by deterioration of other consonants, resulting in a small overall effect of NLFC on consonant recognition. The asymmetrical confusion pattern between alveolar sounds and retroflex sounds as well as greater confusion as the compression setting changed from SR2 default to SR2 strong was consistent with the acoustic change caused by frequency compression (Yang et al., 2018). The acoustic energy of retroflex sounds /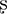, t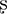, t
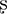
^h^/ (sh, zh, ch) concentrates at a lower frequency region that was less affected by frequency compression. By contrast, the alveolar sounds /s, ts, ts^h^/ (s, z, c) have spectral energy distributed at a higher-frequency region. Frequency compression shifted the spectral energy of alveolar sounds to a lower-frequency region similar to retroflex sounds. The more aggressive compression setting caused greater distortion of spectral energy distribution and thus greater confusion. While both alveolar and alveolopalatal sounds are high-frequency sounds that present accessibility challenges in people with severe hearing impairments, the higher accuracy and less confusion of alveolopalatal sounds were likely associated with the phonotactic constraints in Mandarin Chinese. Mandarin /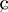, t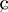, t
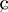
^h^/ (x, j, q) is always followed by /i/, /y/, or vowels starting with these two. In the current study, the alveolopalatal sounds were followed by /ia/ which differed from other tested consonants that were followed by /a/. The distinct vowel environment likely assisted in the recognition by the hearing aid users.

Although adaptive NLFC technology helped improve patients’ consonant recognition and audibility of high-frequency information in quiet, it did not provide significant perceptual benefits to sentence recognition in noise. Previous research revealed mixed findings on sentence recognition in noise with the NLFC technology (Glista et al., 2009; Perreau et al., 2013; Bentler et al., 2014; McCreery et al., 2014; Picou et al., 2015). Chen et al. (2020) reported improved sentence recognition accuracy in patients with SR on than with SR off. In the present study, sentence recognition performance did not show significant change among different SR2 settings. However, similar to Chen et al. (2020), the participants in the present study demonstrated substantial individual differences in sentence recognition. While some participants (8/29) showed a benefit of more than 5 percentage points with SR2 default or SR2 strong setting than SR2 off across all five sessions; some (8/29) had a decreased accuracy of more than 5 percentage points; the others (13/29) demonstrated a small change less than 5 percentage points. Varying degrees of hearing loss (from moderate to profound) in the 29 participants could result in different optimal compression situations. However, our correlational analyses did not find a correlation between the aided thresholds and the amount of NLFC benefits in speech recognition. Cognitive ability and level of linguistic knowledge could also potentially affect sentence recognition in noise. Although age is not the only factor related to cognitive ability and level of linguistic knowledge and we found no correlation between age and speech recognition performance in our sample, other studies have indicated that those factors might be especially important for elderly patients with profound hearing loss (Best et al., 2018; Nuesse et al., 2018; Vermeire et al., 2019). Another note is that the sentence materials used in this study were recorded by a male talker. Our data suggested that female voice tended to receive higher sound-quality ratings than the male voice. Wang and Xu (2020) also showed that Mandarin tone recognition in noise using a female voice yielded higher scores than using a male voice. It would be interesting to examine whether female voice would lead to a greater improvement for sentence recognition in NLFC conditions.

Sound quality is another core criterion in evaluating patients’ willingness to accept technology and degree of comfort after using it. Some researchers reported no obvious changes in sound-quality ratings with or without NLFC (Uys et al., 2012; Parsa et al., 2013; Kirchberger and Russo, 2016; Tseng et al., 2018; Chen et al., 2020). Some researchers found that patients with moderate to severe sensorineural hearing loss showed a preference for listening to music with NLFC scheme (Uys et al., 2016). As reported in Xu et al. (2020) who used the same test materials for sound-quality ratings as the present study, no deterioration of sound quality was found between stronger settings of SR2 and SR2 off setting, which suggested a great tolerance to the adaptive NLFC. Consistent results were found in the present study. In general, participants favored SR2 default setting over SR2 off or SR2 strong settings (Figure 8). Furthermore, our study demonstrated that patients generally favored female voice over the other four types of materials (i.e., own voice, male voice, chirp, music) in the four perceptual categories of loudness, clarity, naturalness, and overall quality rating. By contrast, own voice had the least favored sound quality, at least for the first three sessions. Such poor rating in “own voice” is usually attributed to the occlusion effects caused by the hard ear-molds used in the present study. However, we have no direct evidence of perceived occlusion effect in our participants. It was worth noting that through periods of adaption, our participants rated their own voice significantly better in the fifth session than in their first three sessions, which indicated the importance of acclimatization for the SR2 function as further elaborated below.

Researchers have found that a period of acclimatization is necessary for the human brain to gradually learn and obtain benefits from any newly applied technologies including the NLFC scheme. Giroud et al. (2017) found that it took several weeks for hearing aid users to acclimate themselves to the new hearing aid algorithm of NLFC. Glista et al. (2009) reported that the average time for patients to adapt to the NLFC processor was approximately 10 weeks. In the present study, all participants had a period of 12 weeks to adapt to the NLFC scheme. Test session was found to be the main factor for vowel, consonant, sentence recognition, and sound-quality ratings. It should be noted that no interaction of test session by SR2 setting was evident. This suggested that the improvement might be the outcome of perceptual learning or training effects (i.e., participants’ increased familiarity with the test materials and procedure) instead of auditory acclimatization, *per se*. This finding was consistent with the previous SR study (Chen et al., 2020). The present study only examined speech-recognition performance and subjective ratings for 3 months. On one hand, we observed continuous improvement in the first few weeks, indicating that the participants adapted to the new hearing aids in a very short period. On the other hand, had we extended the observation to a longer period, we might see further continuous improvement in both speech recognition and subjective quality ratings. Future studies will be necessary to demonstrate the long-term benefits of using the NLFC technology in hearing-impaired listeners. Further, one limitation of the present study is the lack of a control condition in which the SR2 off setting is used in the hearing aids for the same amount of time as the SR2 default and SR2 strong settings. With such a control condition, we would be in a better position to evaluate whether the perceptual benefits (such as in consonant recognition task and overall sound-quality ratings) resulted from a training effect or from the application of the SR2 technology itself.

## Conclusion

In summary, the adaptive NLFC technology implemented the hearing aids provided benefit to high-frequency sound detection, as well as Mandarin consonant recognition, and sound-quality ratings in listeners with moderate-to-profound sensorineural hearing loss. As expected, vowel recognition performance with or without the adaptive NLFC algorithm was consistently good, indicating no deterioration of vowel perception using the adaptive NLFC. However, for sentence recognition in noise, the adaptive NLFC algorithm showed limited effects. Among different settings, SR2 default provided more benefits than SR2 strong setting in our group of participants with moderate to profound hearing loss. Furthermore, participants showed continuous improvement of recognition performance with increased length of SR2 use, which indicated a potential effect of auditory acclimatization.

## Data Availability Statement

The raw data supporting the conclusions of this article will be made available by the authors, without undue reservation.

## Ethics Statement

The studies involving human participants were reviewed and approved by the Institutional Review Boards of Ohio University and Beijing Tongren Hospital. The patients/participants provided their written informed consent to participate in this study.

## Author Contributions

XC, JR, VK, and LX conceived and designed the study. SQ, XT, HH, and JG collected the data. XW performed the statistical analyses. SQ, JY, and LX wrote the manuscript. All authors discussed the results and implications and commented on the article at all stages.

## Conflict of Interest

XT, HH, and JG were employed by the company Sonova China, Shanghai. JR and VK were employed by the company Sonova AG Switzerland. The remaining authors declare that the research was conducted in the absence of any commercial or financial relationships that could be construed as a potential conflict of interest.

## Publisher’s Note

All claims expressed in this article are solely those of the authors and do not necessarily represent those of their affiliated organizations, or those of the publisher, the editors and the reviewers. Any product that may be evaluated in this article, or claim that may be made by its manufacturer, is not guaranteed or endorsed by the publisher.
